# Pluripotent Stem Cells for Spinal Cord Injury Repair

**DOI:** 10.3390/cells10123334

**Published:** 2021-11-27

**Authors:** Maria Martin-Lopez, Beatriz Fernandez-Muñoz, Sebastian Canovas

**Affiliations:** 1 Cellular Reprogramming and Production Unit, Andalusian Network for the Design and Translation of Advanced Therapies, 41092 Sevilla, Spain; beatriz.fernandez.munoz@juntadeandalucia.es; 2 Physiology of Reproduction Group, Physiology Department, Mare Nostrum Campus, University of Murcia, 30100 Murcia, Spain; 3 Biomedical Research Institute of Murcia, IMIB-Arrixaca-UMU, 30120 Murcia, Spain

**Keywords:** tetraplegia, cell therapy, animal models, PSC, ESC, iPSC, NSC

## Abstract

Spinal cord injury (SCI) is a devastating condition of the central nervous system that strongly reduces the patient’s quality of life and has large financial costs for the healthcare system. Cell therapy has shown considerable therapeutic potential for SCI treatment in different animal models. Although many different cell types have been investigated with the goal of promoting repair and recovery from injury, stem cells appear to be the most promising. Here, we review the experimental approaches that have been carried out with pluripotent stem cells, a cell type that, due to its inherent plasticity, self-renewal, and differentiation potential, represents an attractive source for the development of new cell therapies for SCI. We will focus on several key observations that illustrate the potential of cell therapy for SCI, and we will attempt to draw some conclusions from the studies performed to date.

## 1. Introduction

Spinal cord injury (SCI) is a devastating multifactorial event that affects approximately 39 cases per 1 million individuals in North America, with enormous healthcare costs. Cervical lesions, which in most cases result in tetraplegia, are the most common type of lesion, representing 60% of all SCI [1]. While rehabilitation, early surgical decompression, and the use of electrical stimulation have made great strides in increasing the quality of life of patients, it is reasonable to say that there is no curative treatment for this condition. Thus, considering the incidence, the poor long-term prognosis of patients with SCI, and the financial healthcare burden, there is an urgent need to develop new strategies to treat SCI. 

Severe SCI damages gray matter neurons and white matter axonal tracts that carry signals to and from the brain, and involves the cellular loss of nervous tissue, demyelination, the generation of an inhibitory environment and glial scar formation. SCI repair therefore requires axonal regeneration, remyelination, the replacement of lost cells (both neuronal and glial cells), and a permissive environment to increase survival and functional integration with host cells [2,3]. Over the last two decades, much has been said about the potential benefits of cell therapy in SCI; nonetheless, with only a few cell-based products in phase I/II clinical trials, cell therapies are still far from becoming the standard care for patients with SCI.

Numerous SCI animal models have been used to test a variety of cell types, including both tissue-specific cells and stem cells. Among the tissue-specific cells, Schwann cells [4], peripheral nerve grafts [5], genetically-modified fibroblasts [6] and olfactory ensheathing cells (OEC) [7] have been used to examine repair capacity (reviewed by [8,9]); however, with the exception of OEC, no categorical improvements have been demonstrated to justify their use in patients. 

The advantages of stem cells over tissue-specific cells are manifold, of which the most appealing is their capacity to self-renew and their potential to differentiate into multiple lineages. Accordingly, various stem cell populations have been tested to repair SCI, including mesenchymal stem cells, neural stem cells (NSC) and pluripotent stem cell (PSC)-derived cells.

Given the immunomodulatory potential of MSC, they have been used to try to mitigate the negative effects of the pro-inflammatory environment found at the lesion site. The results of animal studies with MSC were promising, and several clinical trials have been launched. However, clinical studies have generally failed to achieve significant functional recovery and restore neural circuits, although some positive results have been reported (reviewed in [10]).

NSC also secrete immunomodulatory and neurotrophic factors, and generate neurons and glial cells, opening the possibility of cell replacement; they therefore seem to have better abilities to improve motor function in SCI [11]. NSC of fetal and adult origin have been shown to provide significant motor recovery in SCI models (reviewed in [12]). Procedures for NSC isolation from fetal and adult sources are inefficient, ethically controversial or require invasive procedures, which have obvious limitations. Nonetheless, NSC from the central nervous systems of fetuses are perhaps the most used cell type. In fact, at least two companies are using these cells in clinical trials for SCI (reviewed in [13]).

**Figure 1:**
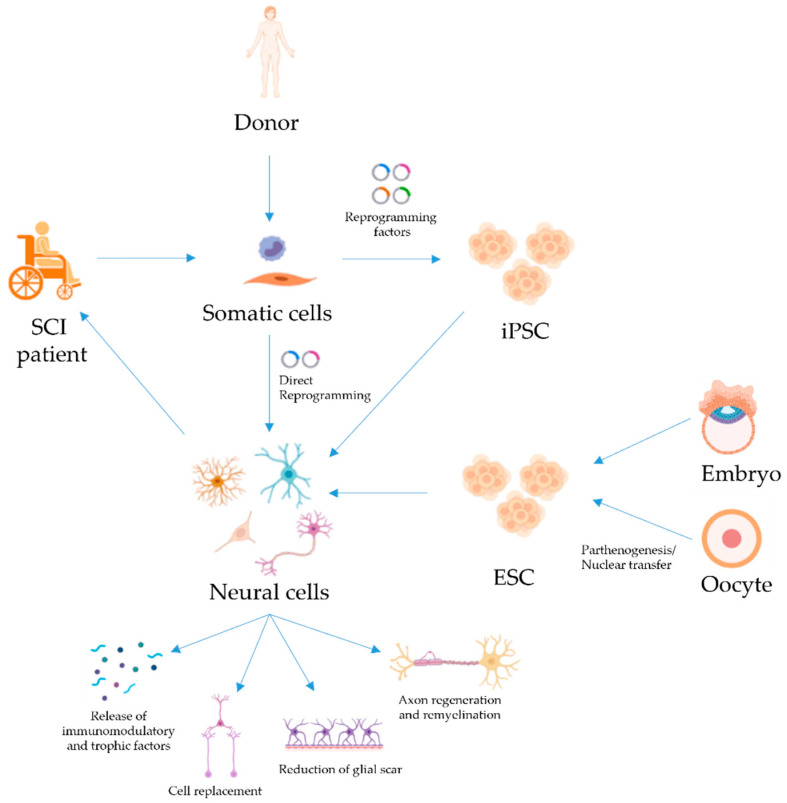
Treatments of SCI with pluripotent and reprogrammed cells. Patient- or donor-derived somatic cells can be reprogrammed into iPSC, which can then be differentiated into neural cells for transplantation to restore function in patients with SCI. PSC derived from embryos or oocytes are an alternative source of neural cells for therapy. Direct reprogramming of somatic cells to neural cells (without passing through a pluripotent state) has also been achieved and could be used for SCI treatment. Several mechanisms for neural cell-mediated repair have been suggested, such as cell replacement, the release of immunomodulatory and trophic factors, regeneration and remyelination of axons, and/or reduction of glial scarring. Created with BioRender.com (last accessed on 15 November 2021).

**Figure 2:**
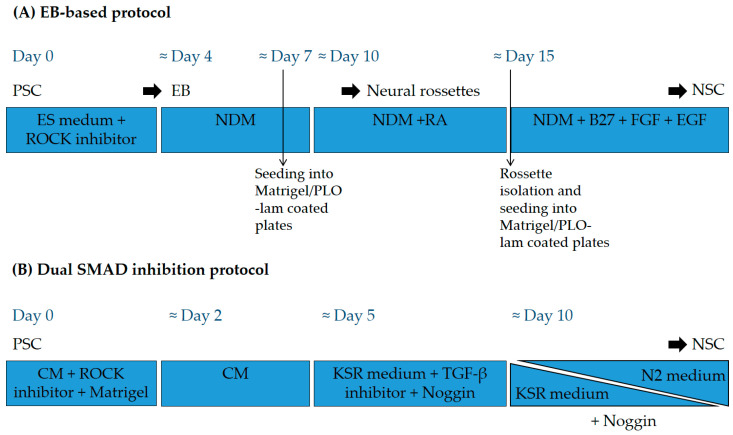
General scheme of differentiation protocols used to produce NSC from PSC. (**A**) Example of an EB-based protocol. Undifferentiated PSC are grown in suspension in embryonic stem (ES) medium with ROCK inhibitor to form embryoid bodies (EB) that are further cultured in neural differentiation medium (NDM) containing N2 supplement, seeded on coated plates, and directed to form neural rosettes containing neuroepithelial cells. (**B**) Example of a dual SMAD inhibition-based protocol. Undifferentiated PSC are plated on Matrigel as single cells in an MEF (mouse embryonic fibroblasts)-conditioned medium (CM) and different inhibitors are added to the cells in the presence of knockout serum (KSR) medium. KSR medium is then gradually replaced by medium with N2 supplement. ROCK: Rho kinase; PLO-lam: poly-L-ornithine-laminin; RA: retinoic acid; FGF: fibroblast growth factor; EGF: epidermal growth factor; TGF-β: transforming growth factor beta.

Pluripotent stem cells (PSC) represent a promising alternative source of stem cells, mainly due to the fact that they have a higher self-renewal and differentiation capacity than more committed stem cell types, albeit with considerable challenges limiting their implementation. PSC are typically obtained from pre-implantation embryos (embryonic stem cells, or ESC) or by reprogramming somatic cells into ESC-like cells (induced pluripotent stem cells, or iPSC), and they have the intrinsic capacity for differentiation into every cell type in the whole organism (Figure 1). Different types of neural cells derived from in vitro cultures of PSC have been tested for SCI repair in multiple studies with varying degrees of success. Although PSC differentiation protocols can differ between studies, in general, NSC are produced from PSC by inducing embryoid body (EB) formation or by dual SMAD inhibition (Figure 2). Further differentiation can be achieved to obtain more mature cell types. Compared to more differentiated cells, PSC-derived NSC have the advantage of allowing a degree of flexibility regarding the final developmental fate of the transplanted cells. Assuming that the current working hypothesis is correct, the niche will dictate the type of cells required, and transplanted NSC would provide not only such differentiated cells, but could also adapt to the variability that exists between lesions in the spinal cord.

Paradoxically, while most researchers agree that PSC-derived cells may offer a permanent therapeutic solution to SCI, after more than 40 years and over a decade since the descriptions of ESC and iPSC, respectively, the adoption of these cell types for the development of novel SCI treatment modalities has been slow. In this review, we focus our analysis on the progress that has been made using PSC-derived cells in an effort to alleviate SCI. Studies wherein PSC have not been differentiated to a more committed cell type and have been directly transplanted into animals and humans have not been considered in this review, as the transplantation of undifferentiated PSC usually leads to tumor formation [14], and thus should not be considered for the development of cell therapies. Studies in which cells were applied in the same surgical procedure in which SCI was performed have not been included in this review, as they do not simulate the reality of the pathology or future therapy in humans.

## 2. Embryonic Stem Cells for SCI Repair

During the last two decades, numerous studies have explored whether the use of ESC-derived cells has some benefit for SCI repair (Table 1). Many studies have differentiated ESC to neural cells with stem properties and have called them neural stem, neural progenitor or neural precursor cells. Since these terms are sometimes used interchangeably, in this review, we only use the term neural stem cells (NSC) to simplify. McDonald and colleagues [15] were the first to evaluate murine ESC differentiated to the neural stem lineage to promote recovery after SCI. Cells transplanted into a rat model of subacute SCI differentiated into astrocytes, oligodendrocytes, and neurons. The preliminary results were encouraging, and rats displayed locomotor recovery by hindlimb weight support and partial hindlimb coordination. However, transplanted cells were poorly characterized—a fact that limited the interpretation of the results. Another pioneering study transplanted murine ESC into the injured area of mice with subacute SCI 10 days post-injury. Compared to the control group, mice transplanted with NSC showed significant score improvements in three behavioral tests. Additionally, neurons and oligodendrocytes were detected in the graft areas; however, few axons penetrated or sprouted from the grafts [16]. These results were encouraging, but the NSC used were poorly characterized, only considering the expression of nestin, hindering the replication of these results. 

ESC-derived neurospheres (NS) containing NSC have also been used for SCI cell therapies. To evaluate its efficacy for SCI repair, Kumagai and colleagues [17] compared the grafting of primary NS (from a single cell suspension of murine EB-derived neurospheres) versus secondary NS (obtained by secondary culture of dissociated primary NS) in subacute SCI (9 days post-injury). Surprisingly, gliogenic secondary NS, but not neurogenic primary NS, promoted axonal growth, remyelination and angiogenesis, and resulted in significant locomotor functional recovery after SCI. In another study, NSC from human ESC, embedded in fibrin matrices containing a growth factor cocktail, were grafted into the injured spinal cord in rats. After complete spinal cord transection, ESC-derived NSC formed large numbers of projections from the injury site. The derived axons expressed the presynaptic marker synatophysin, and the graft-derived axons were myelinated by host oligodendrocytes, suggesting integration with host cells [18].

Another strategy applied for SCI repair has been the use of ESC-derived cells with forced expression of specific factors. For example, Butenschön and colleagues reported the effect of mouse ESC–NSC overexpressing BDNF, isolated by magnetic and fluorescent-activated cell sorting, in subacute SCI. Recovery of motor function was observed only in animals transplanted with SSEA-1-/PSAN-CAM+ cells overexpressing BDNF, but not in control NSC [19]. Another study [20] used substrate adherent ESC-derived neural aggregates constitutively overexpressing the neural cell adhesion molecule L1. Neural aggregates-L1 cells transplanted 3 days after injury rescued endogenous spinal cord interneurons and motor neurons, and promoted the regrowth of catecholaminergic nerve fibers distal to the lesion site. 

In general, the results reported using mouse and human NSC differentiated from ESC could be considered positive (Table 1). In fact, at least one clinical trial has been launched in 2021 with ESC–NSC for cervical sub-acute SCI (NCT04812431) (Table 2). In this trial sponsored by S. Biomedics Co (Seoul, Republic of Korea), two to six subjects with damage at the C4-C7 level will be recruited and administered ESC–NSC PSA-NCAM positive, to evaluate safety and exploratory efficacy. The cells will be administered intrathecally, in five areas, and all subjects will be subjected to a follow-up study after a period of 1 year and 5 months. This clinical trial has been launched very recently and no patient has yet been recruited, but the results obtained from this study could be valuable to determine whether ESC-derived NSC are safe. There is always a potential safety risk when transplanting cells that are not terminally differentiated, and often poorly characterized. Potential ectopic spreading (dissemination over extended distances) is of concern. The high pressure used during NSC injection could force cell egress from the injury site and favor remote cell dissemination. However, the use of another injection method with lower pressure did not show dissemination in monkeys [21,22]. In addition, there are differences between animal models of SCI (mainly rat) and human SCI, including open versus closed lesions, and the fact that the human vestigial central canal is functionally closed in most individuals by the second decade of life. These dissimilarities would suggest that the risk of biodistribution would be lower in humans [21,22]. Clearly, studies are warranted to resolve these issues.

Assuming that all injuries in the spinal cord are created equally, some investigators have chosen to transplant cells that are further along the differentiation path than NSC, including oligodendrocytes, astrocytes, and a variety of neuronal-type cells. This assumption entails a significant speculation considering the multiple tracks involved and the different causes of trauma in SCI. The obvious advantages of this approach are the fact that the tumorigenic potential will probably decrease, and that it allows some degree of control over the final cell types that are grafted, albeit at the expense of having to speculate the type of cells each particular lesion needs. 

In this regard, the first study to analyze the therapeutic effect of more differentiated cells used human ESC-oligodendrocyte progenitor cells (OPC) in a rat model of spinal cord injury [23]. After grafting these OPC into rats with subacute and chronic injuries (7 days or 10 months after injury, respectively), only those animals that received the transplant in the subacute phase showed enhanced remyelination and improved motor function. The same group [24] tested the therapeutic effect of human ESC–OPC using a moderate and severe contusive spinal cord injury model. The severe contusion induced extensive demyelination, and the transplantation of human ESC–OPC showed robust remyelination. Based on the efficacy results shown in the preclinical studies and on the extensive safety analyses [25], the Food and Drug Administration (FDA) approved a phase I clinical trial in 2009 (NCT01217008) sponsored by Geron Corporation (Menlo Park, CA, USA), aimed at analyzing the safety of human ESC–OPC (GRNOPC1) [23,24,26]. The trial was halted for reasons other than those related to safety or efficacy. Full data on the outcome of these experiments have not yet been published [25], but some information was shared in various scientific forums. It was reported that no serious adverse events were detected in the first five patients that received GRNOPC1 at a low dose (2 million cells). The only side effects observed were related to the immunosuppressive regime used (tacrolimus). No changes in the spinal cord or neurological condition were found, and while the cells used were allogeneic, there was no apparent evidence of immunological rejection. At the end of 2013, Geron’s Stem Cell Program was taken over by Asterias Biotherapeutics, Inc. (Fremont, CA, USA), and GRNOPC1 was renamed AST-OPC1. The new strategy advanced by Asterias was a dose-escalating trial to treat three patients with cervical injuries using a low dose of cells, and subsequently to treat more patients with higher doses to assess whether the therapy could restore any sensory and/or motor function in the trunk and/or limbs [26]. In August 2015, with financial support from the California Institute for Regenerative Medicine, as a strategic partnership award, Asterias relaunched a phase I/II open-label clinical trial (NCT 02302157), and the initial low-dose (2 million cells) safety cohort, which included three patients. In subsequent phases, they tested sequentially increasing doses of 10 to 20 million cells in one or two injections, to be administered 21 to 42 days after injury in 22 patients with subacute, C-5 to C-7, neurologically complete SCI. Preclinical efficacy and safety data in a nude rat model of cervical SCI showed improved locomotor performance, using the automated TreadScan system, when human AST-OPC1 were administered directly into the cervical spinal cord in subacute injury, and no adverse effects were reported [27]. Since 2019, when BioTime acquired Asterias, creating the cell therapy company Lineage Cell Therapeutics, manufacturing has been completely transferred to the company’s current Good Manufacturing Practice (cGMP) facility in Israel, where key process improvements have been developed and implemented. According to the available information from the company, after one year, 96% of the treated patients reported improved motor function (one-third of the patients gained two levels of motor function and two-thirds gained one level) [28,29]. 

Human ESC–OPC have also been tested in a cervical rat model, rather than the more commonly used thoracic model [30]. Grafted cells attenuated the severity of the injury and improved the recovery of forelimb function and range of motion. The histological effects of transplantation included robust white and gray matter sparing at the injury epicenter, and specifically, preservation of motor neurons that correlated with movement recovery. They also identified gene expression changes supporting the histological and functional improvement. Another group assayed the effect of allogeneic ESC–OPC transplantation in the subacute phase of cervical SCI in marmosets, a non-human primate. The grafted cells survived and showed the potential to differentiate into the three neural lineages in the injured spinal cord environment. Derived oligodendrocytes contributed to the remyelination of axons, and synaptic connections between grafted green fluorescent protein (GFP)-positive neurons and host cells were observed. These facts support the motor functional recovery observed. In addition, regarding safety, the results show that the marmoset recipient lymphocytes did not respond to allogeneic ESC-derived cells, and no signs of tumorigenicity after transplantation were observed [31]. 

Mouse ESC-derived glial progenitors positive for the nerve glial antigen 2 (NG2) marker and matrix metalloproteinase 9 have also been tested. These cells could penetrate the glial scar formed after subacute SCI. In addition, the axons of these cells grew over long distances (>10 mm) with a preference to traverse white matter rather than gray matter. These facts support the notion that the expression of chondroitin sulfate proteoglycan in the injury scar is an impediment to regeneration, and that NG2-positive ESC-derived glial progenitors can breach this barrier and promote axon growth [32].

Allodynia and hyperalgesia are the two forms of spontaneous neuropathic pain that affect approximately 50% of SCI patients and persist over time, complicating the rehabilitation and decreasing the quality of life. Hwang and colleagues, in an effort to address this problem, transplanted ESC-derived spinal GABAergic neural precursors, 3 weeks post-injury. They observed a reduction in neuropathic pain in injured animals 2 weeks post-transplantation, and the effect persisted for up to 7 more weeks, although locomotor function did not improve [33]. Fandel and colleagues also showed that human ESC-derived inhibitory interneuron precursors were able to substantially ameliorate neuropathic pain and improve bladder function, although they did not find a noticeable locomotor recovery [34].

In this context, other groups have tested a combination of cells. For example, Niapour and colleagues transplanted a combination of Schawnn cells isolated from the sciatic nerve and human ESC–NSC, with the goal of improving the differentiation of NSC after transplantation, into a subacute SCI rat model at thoracic level [35]. The presence of Schawnn cells in the human ESC–NSC + Schawnn cells-transplanted group was found to significantly boost the proportion of neuronal markers (TUJ1 and MAP2). Although enhanced locomotor function recovery was observed in all groups (NSC, Schawnn cells, NSC + Schawnn cells), a synergistic effect was promoted by the co-transplantation of human ESC–NSC and Schawnn cells. Animals receiving co-transplants established a better state, as assessed by the BBB functional test at week 5. Similarly, Salehi and co-workers [36] used the co-transplantation of ESC-derived motor neurons and OEC in a subacute thoracic SCI in rats, using the Vanicky’s method for SCI [37], which may not be sufficiently precise and reproducible. The co-transplantation of ESC-derived motor neurons and OEC also had a synergistic effect in promoting neural regeneration and survival, but the recovery of hindlimb function was not significantly enhanced by co-transplantation. Taken together, these three studies—while still far from explaining the mechanism of action responsible for the observed recovery in transplanted animals—highlight the potential of using a combination of different, and more differentiated, cell types for transplantation. Nevertheless, the specific combination (and percentage) of each cell type remains to be determined. 

To date, the studies performed with ESC-derived cells have shown, in general, that these cells can be efficacious in acute SCI models (Table 1), but further optimization is needed to develop efficacious therapies in humans. Furthermore, the tumorigenic potential of ESC-derived products has been evidenced in some studies. Special care should be taken to ensure that no undifferentiated ESC contaminate the final product, and to verify the safety of the final cell product, especially when using non-terminally differentiated cells. Another important drawback to the use of ESC is the ethical concerns related to the use of embryonic tissue (reviewed in [38]). Nevertheless, there are several clinical trials on-going with ESC-derived cells for SCI and other pathologies [39]. Results from these clinical trials will probably provide relevant data about the safety and scalability of ESC-derived cells. 

**Table 1:** Selected studies carried out with PSC-derived cells for acute and chronic SCI.

SCI Phase	Type of SCI	Animal Model	Level of Injury	Injected Cells	Number of Cells	Application Route	Timing of Transplantation	Tests Used for the Assessment of Recovery	Outcome	Year	Reference
Acute	Contusion	Rat	Thoracic	Mouse RA-differentiated ESC–NSC	1 × 10^6^	At the lesion epicenter	9 days PI	BBB	Locomotor recovery	1999	[15]
	Contusion	Rat	Thoracic	Human ESC–OPC	2.5 × 10^5^ or 1.5 × 10^6^	Rostral and caudal to the lesion epicenter	7 days PI	BBB and 4 parameter kinematic analyses	Locomotor recovery	2005	[23]
	Contusion	Mouse	Thoracic	Mouse ESC–NSC	2 × 10^4^	At the lesion epicenter	10 days PI	Motor score, platform hang and rope walk	Locomotor recovery	2005	[16]
	Contusion	Rat	Thoracic	Human ESC–OPC	1.5 × 10^6^	Rostral and caudal to the lesion epicenter	7 days PI	BBB	Transplantation per se did not decrease locomotor function	2006	[24] ^#^
	Contusion	Mouse	Thoracic	Mouse ESC–primary and secondary neurospheres	5 × 10^5^	At the lesion epicenter	9 days PI	BMS	Locomotor recovery	2009	[17] *
	Compression	Rat	Thoracic	Mouse ESC-motorneurons + OEC	1 × 10^6^	At the lesion epicenter	9 days PI	BBB	Locomotor recovery	2009	[36]
	Contusion	Rat	Cervical	Human ESC–OPC	1.5 × 10^6^	Rostral and caudal to the lesion epicenter	7 days PI	Forelimb movement scores	Locomotor recovery	2010	[30]
	Compression	Mouse	Thoracic	Mouse ESC–neural aggregates overexpressing L1	2 × 10^5^	Rostral and caudal to the lesion epicenter	3 days PI	BBB score, foot-stepping angle and rump-height index	Locomotor recovery	2011	[20]
	Contusion	Rat	Thoracic	Human ESC–NSC + Schwann cells	5 × 10^5^ (NSC or Schwann cells) or 1 × 10^6^ (NSC + Schwann cells)	At the lesion epicenter	7 days PI	BBB	Locomotor recovery	2012	[35]
	Transection	Rat	Thoracic	Human ESC–NSC in fibrin with a growth factor cocktail	2 × 10^6^	At the lesion epicenter	7 days PI	BBB, 21-point locomotion rating scale, electrophysiological assessment	Locomotor recovery	2012	[18]
	Contusion	Rat; mouse	Thoracic	Human ESC–OPC	Rats: 2.4 × 10^5^ or 2.4 × 10^6^. Mice: 2.5 × 10^5^ to 1 × 10^6^	Rats: 4 injections at the perimeter of the lesion or 1 injection rostral to the lesion epicenter. Mice: rostral to the lesion epicenter.	6–8 days PI	None	Locomotor recovery in previous studies (see [23,24])	2015	[25]
	Contusion	Rat	Thoracic	NG2 and MMP9 positive mouse ESC–NSC	1 × 10^6^	At the lesion epicenter	9 days PI	None	Axonal outgrowth into white matter	2015	[32]
	Contusion	Marmoset	Cervical	OPC-enriched marmoset ESC–NSC	1 × 10^6^	At the lesion epicenter	14 days PI	Open field and bar grip strength test	Locomotor recovery	2015	[31] *
	Contusion	Mouse	Thoracic	Human ESC-derived inhibitory interneuron precursors	3 × 10^5^ or 6–8 × 10^5^	Caudal to the injury epicenter	15 days PI	BMS, Allodynia, Thermal hyperalgesia and bladder functional tests	Absence of locomotor recovery	2016	[34]
	Contusion	Mouse	Thoracic	Mouse ESC–NSC overexpressing BDNF	1 × 10^5^	At the lesion epicenter	7 days PI	BMS	Locomotor recovery	2016	[19]
	Contusion	Rat	Thoracic	Mouse ESC–NSC	1 × 10^6^	At the lesion epicenter	21 days PI	BBB and CBS	Absence of locomotor recovery	2016	[33]
	Contusion	Mouse	Thoracic	Murine iPSC and ESC–primary and secondary neurospheres	5 × 10^5^	At the lesion epicenter	9 days PI	BMS	Locomotor recovery	2010	[40] *
	Contusion	Rat	Thoracic	Mouse iPSC–derived astrocytes	1 × 10^5^	At the lesion epicenter	3–7 days PI	BBB, inclined-plane test, SCANET MV-40, sensory tests	Absence of locomotor recovery	2011	[41]
	Contusion	Marmoset	Cervical	Human iPSC–secondary and tertiary neurospheres	1 × 10^6^	At the lesion epicenter	9 days PI	Open field, bar grip, and cage climbing tests.	Locomotor recovery	2012	[42] *
	Contusion	Mouse	Thoracic	Human iPSC–NSC	1 × 10^6^	At the lesion epicenter	7 days PI	BMS, MEPs	Locomotor recovery	2012	[43]
	Hemisection	Rat	Cervical	Human iPSC–NSC in a fibrin matrix and growth factor cocktail	1.25 × 10^6^	Three pairs of injections 0.5 mm apart, at the center, rostral, and caudal to the center of the lesion cavity	14 days PI	Grid-walking, forelimb grooming and LUAT	Absence of locomotor recovery	2014	[44]
	Compression	Rat	Thoracic	Human iPSC–NSC	5 × 10^5^	At the lesion epicenter	7 days PI	BBB, plantar test, beam walking test, and RotaRod	Locomotor recovery	2015	[45]
	Compression	Mouse	Thoracic	Mouse iPSC–NSC	2 × 10^5^	Four injections flanking the injury	7 days PI	BMS, CatWalk, mechanical and thermal allodynia tests.	Locomotor recovery	2015	[46]
	Contusion	Mouse	Thoracic	Murine iPSC-derived neurospheres	5 × 10^5^	At the lesion epicenter	9 days PI	BMS, RotaRod and DigiGait	Locomotor recovery	2011, 2015	[47,48] *
	Contusion	Mouse	Thoracic	Human iPSC–NSC	4 × 10^5^	Rostral and caudal to the lesion epicenter	7 days PI	BMS and Catwalk	Absence of locomotor recovery	2015	[49]
	Compression	Rat	Thoracic	Human iPSC–NSC	5 × 10^5^	At the lesion epicenter	7 days PI	BBB, plantar test, beam walking test and RotaRod	Locomotor recovery	2015	[50]
	Compression	Rat	Thoracic	Human iPSC-OPC in a hydrogel with RGD and PDGF-A	8 × 10^5^	Rostral and caudal to the lesion epicenter	7 days PI	BBB	Locomotor recovery	2016	[51]
	Contusion	Mouse	Thoracic	Human iPSC–OPC	5 × 10^5^	At the lesion epicenter	9 days PI	BMS, RotaRod and DigiGait	Locomotor recovery	2016	[52] *
	Contusion	Mouse	Thoracic	Human iPSC–NSC treated with γ-secretase inhibitor	5 × 10^5^	At the lesion epicenter	9 days PI	BMS	Locomotor recovery	2016	[53] *
	Contusion	Mouse	Thoracic	Human iPSC–NSC	5 × 10^5^	At the lesion epicenter	9 days PI	BMS	Locomotor recovery (declined when tumors formed)	2017	[54] *
	Compression	Rat	Thoracic	Human iPSC–NSC conditioned with EI-tPA	1.5 × 10^6^	At the lesion epicenter	7 days PI	BBB	Locomotor recovery	2019	[55]
	Contusion	Mouse	Thoracic	Human iPSC–spinal cord–NSC	5 × 10^5^	At the lesion epicenter	9 days PI	BMS, RotaRod and treadmill analysis	Locomotor recovery	2020	[56] *
	Contusion	Mouse	Thoracic	Human iPSC–NSC	1 × 10^5^	Rostral to the lesion epicenter	7 days PI	BMS	Locomotor recovery	2021	[57]
	Contusion	Rat	Thoracic	Human iPSC–NSC + MSC + PA-C	1.8 × 10^6^	Rostral, caudal, and at the lesion epicenter	7 days PI	BBB and Catwalk	Absence of locomotor recovery	2021	[58]
Chronic	Contusion	Rat	Thoracic	Human ESC–OPC	2.5 × 10^5^ or 1.5 × 10^6^	Rostral and caudal to the lesion epicenter	10 months PI	BBB and four-kinematic analyses	Absence of locomotor recovery	2005	[23]
	Contusion	Rat	Cervical	Human iPSC–NSC	2 × 10^5^	Rostral and caudal to the lesion epicenter	30 days PI	LUAT, FRT, allodynia test	Absence of locomotor recovery	2013	[59]
	Contusion	Mouse	Thoracic	Human iPSC–NSC treated with γ-secretase inhibitor	5 × 10^5^	At the lesion epicenter	42 days PI	BMS, RotaRod and treadmill analysis	Locomotor recovery	2018	[60] *
	Compression	Rat	Thoracic	Human iPSC–NSC on Laminin-Coated pHEMA-MOETACl Hydrogel	3 × 10^5^	At the lesion epicenter	35 days PI	BBB, plantar test	Absence of locomotor recovery	2019	[61]
	Accidental SCI	Dog	Thoracic	Canine iPSC–NSC	2 × 10^6^	At the lesion epicenter, and one vertebral space caudal and rostral to the lesion	>28 days PI	Neurological and electrophysiological evaluation	Absence of locomotor recovery	2020	[62]
	Contusion	Rat	Thoracic	Glial scar photo-ablation + iPSC–regionally specific spinal pre-OPC	5 × 10^5^	Rostral and caudal to the lesion epicenter	70 days post-injury	BBB	Absence of locomotor recovery	2021	[63]
	Contusion	Rat	Cervical	Human iPSC–NSC	4 × 10^5^	Rostral and caudal to the lesion epicenter	28 days PI	FRT, IBB, and LUAT	Absence of locomotor recovery	2021	[64]

SCI: spinal cord injury; PI: post-injury; RA: retinoic acid; ESC: embryonic stem cells; NSC: neural stem cells; OPC: oligodentrocyte progenitor cells; OEC: olfactory ensheathing cells; NG2: nerve glial antigen 2; MMP9: matrix metalloprotease 9; BDNF: brain-derived neurotrophic factor; RGD: arginine–glycine–aspartate peptide: PDGF-A: platelet-derived growth factor A; EI-tPA: enzymatically inactive tissue-type plasminogen activator; MSC: mesenchymal stem cells; PA-C: pH-responsive polyacetal–curcumin nanoconjugate; pHEMA: poly(2-hydroxyethyl methacrylate); MOETACl: co-monomer (2-(methacryloyloxy) ethyl trimethylammonium chloride; BBB: Basso, Beattie, and Bresnahan test; BMS: Basso mouse scale; CBS: combined behavioral score; MEPs: motor-evoked potentials; LUAT: limb-use asymmetry test; FRT: forelimb reaching task; IBB: Irvine, Beatties and Bresnahan test; GABA: Gamma aminobutyric acid. ^#^ Keirstead Laboratory; * Okano Laboratory.

## 3. iPSC for SCI Repair

iPSC are artificially induced pluripotent stem cells from adult somatic cells (Figure 1), overcoming the ethical problems associated with the use of embryonic stem cells. Several groups, including our own, have evaluated the transplant of iPSC-derived cells in preclinical models of SCI (Table 1). Similarly to ESC, many researches chose iPSC-derived NSC to treat SCI. For example, Fujimoto and colleagues showed that iPSC–NSC have a therapeutic potential comparable with NSC isolated from human fetal spinal cord in a mouse acute model of thoracic SCI. Furthermore, the iPSC–NSC group showed enhanced remyelination and axon regeneration, and supported the survival of endogenous neurons. Motor function recovery was promoted through the reconstruction of the corticospinal tract, restoring disrupted neuronal circuitry in a relay manner. In addition, these authors used specific cell ablation protocols with diphtheria toxin. After the recovery of motor function was observed, diphtheria toxin was administered to the transplanted animals and, as expected, the condition of the animals worsened, demonstrating that recovery was attributed to transplanted cells [43]. 

Romanyuk and co-workers also reported the beneficial effects of human iPSC–NSC in a rat model using balloon-induced acute SCI at the thoracic level [45]. Cells were transplanted in the acute phase and showed robust survival, and migrated and partially filled the lesion cavity, resulting in significant motor improvement from the second week after transplantation. Similarly, human iPSC–NSC were transplanted into a rat model using the same conditions with the intention of testing the administration route. Cells were transplanted intrathecal and intraspinally, finding, in both cases, that the animals improved locomotor function [50]. Another, more recent study reported positive results. Kong and coworkers showed that human iPSC–NSC reduced pro-inflammatory cytokine levels after SCI, evidenced by the reduced glial and fibrotic scar formation, in an acute mouse model of thoracic SCI. The cells, furthermore, promoted the recovery of limb function [57].

Nevertheless, motor recovery is not always achieved in iPSC-derived cell transplantation studies for SCI repair. Many studies have shown that transplanted cells survived and even differentiated into the three lineages in the host tissue, but this does not always imply integration or locomotor improvement. For example, Pomeshchik and colleagues [49] performed thoracic contusions in mice and transplanted human iPSC–NSC 7 days after injury (subacute). Motor function was assessed at 6 weeks post-injury by BMS and Catwalk gait analysis. The authors concluded that transplanted human iPSC–NSC had no effect on the size of the lesion and did not promote behavioral recovery after SCI. Additionally, human iPSC–NSC showed limited long-term survival. The authors attributed these results to an insufficient immunosuppression dose.

In another fashion, the use of regionally specific iPSC–NSC has also been attempted. Kajikawa and coworkers hypothesized that, given that there are subtypes of NSC regarding their regional identity, spinal cord-type NSC would be the most adequate to inject into SCI for regenerative purposes. They injected forebrain and spinal cord-type iPSC–NSC into a mouse acute model of SCI. Both types of NSC were grafted onto host tissue; however, only the spinal cord type showed connection with the corticospinal tract of the host and, moreover, resulted in recovery of motor function [56].

The level at which the SCI is located is an important factor to take into account in terms of achieving recovery in motor function, as cervical lesions entail a much more severe impairment than thoracic ones. The chronicity is also a relevant factor that should be considered. The maturation of the glial scar surrounding the lesion around 2–3 weeks post-injury is well documented to become a barrier to axonal regrowth and tissue regeneration due to the inhibitory environment within the lesion [63,65]. Thus, the moment in which the cell treatment is applied is also critical to the outcome. 

With the aim of modeling the clinically relevant condition of chronic cervical contusion, Nutt and coworkers tested human caudalized iPSC–NSC in a rat cervical model of chronic SCI. They chose to use the Forelimb Reaching Test (FRT), a very stringent test that offers advantages over others, as it provides a more objective behavioral comparison between and within treatment groups [66]. Human iPSC–NSC were delivered to a unilateral cervical contusion injury generated on the dominant forelimb side. Grafted human iPSC–NSC differentiated into neurons, astrocytes, and oligodendrocytes. In addition, mature and specific neuronal subtypes were generated and projections were observed surrounding host neurons, suggesting integration with host networks [59]. However, the study fell short of producing significant functional recovery. This and other studies (Table 1) indicate that further improvements are needed to treat the chronic disease.

In a different approach, some investigators have opted to use scaffold structures as a cell delivery platform, and to expedite cell differentiation and tissue formation. As an example, Lu and colleagues [44] embedded human iPSC–NSC in fibrin matrices containing a growth factor cocktail in cervical subacute models of SCI, both in rats and mice. They found that host serotonergic axons penetrated human iPSC–NSC grafts and expressed the terminal presynaptic marker synaptophysin. Reticulospinal motor axons also penetrated human iPSC–NSC grafts, demonstrating that reciprocal connections had formed from host-to-graft and graft-to-host. However, collagenous rifts, which axons were unable to cross, were present within the centers of most grafts, and the evaluation of locomotor function found no recovery in this regard. More recently, Ruzicka and coworkers used laminin-coated polymer-based hydrogels containing two sizes of pores: large-sized ones, suitable for cell adhesion and expansion, and small-sized pores, which enabled nutrient diffusion. These scaffolds, loaded with iPSC–NSC, were transplanted into a rat model of chronic SCI. Notably, the constructs integrated in the host tissue, but they failed in restoring motor function [61]. 

Combinatorial therapies have also been applied. Some studies have chosen to condition cells with small molecules to improve their performance in vivo. For example, Shiga and collaborators conditioned iPSC–NSC with enzymatically inactive tissue-type plasminogen activator (EI-tPA), prior to grafting into an acute rat model with severe thoracic SCI. EI-tPA interacts with cellular receptors to mediate changes in cell physiology potentially relevant to the challenges of stem cell therapy; it is neuro-protective to cortical neurons, and promotes neurite outgrowth in neurons and neuron-like cells by activating cell-signaling factors, such as c-Src and ERK1/2. Furthermore, it may regulate innate immunity by suppressing toll-like receptor responses. Notably, they found that the cells differentiated, acquired markers of motor neuron maturation, and extended βIII-tubulin-positive axons several spinal segments below the lesion. Furthermore, they observed a decrease in muscle atrophy, and animals had significantly improved motor function, without exacerbating pain [55]. Bonilla and coworkers combined human iPSC–NSC, MSC and a pH-responsive polyacetal–curcumin nanoconjugate (PA-C) that allowed the sustained release of curcumin to treat thoracic SCI in a rat subacute model. This molecule reduces neuroinflammation after SCI by suppressing the TLR4/NF-κB signaling pathway. Furthermore, given the antioxidant and immunomodulatory properties of curcumin, they hypothesized that PA-C pre-treatment could protect iPSC–NSC exposed to cytotoxic doses of hydrogen peroxide. They reported beneficial outcomes, such as the preservation of neuronal fibers and the reduction of scar tissue. Unfortunately, these significant results did not translate into locomotor recovery [58].

In the last few decades, SCI in the elderly population has increased substantially due to life expectancy [1,67]. In this regard, some studies have arisen to model the condition [68]. Our own laboratory has recently published a study that models chronic cervical SCI in aged rats that were further treated with iPSC–NSC. The animals experimented on showed very high mortality rates, both at SCI induction and with iPSC–NSC treatment, due to their age-related frailty, even though we found that the transplanted cells survived for one month in the spinal cord of aged animals, and no signs of tumor development or adverse reactions were noted. Nevertheless, no locomotor improvement was observed after transplantation [64].

An important issue at stake is the safety of iPSC-derived cells, which must be rigorously evaluated before they enter the clinic [69]. Several studies have been published with the aim of finding a process to try to remove tumorigenic cells before or after transplantation. Many of them have been performed by the Okano Laboratory group. They prescreened murine iPSC-derived cells prior to transplantation, and tested their safety in intact mouse intact cords and their efficacy in a subacute contusion injury model [40]. In this study, iPSC colonies were prescreened prior to transplantation, and “safe” colonies properly differentiated and promoted locomotor recovery, while “unsafe” colonies generated teratomas and failed to promote long-lasting locomotor recovery. Given the previous experience of the group with ESC–NSC transplant [17], where secondary NS provided therapeutic benefits, they transplanted cells isolated from both primary and secondary NS. iPSC–secondary NS contributed to remyelination and induced the axonal regrowth of host serotonergic fibers, which resulted in locomotor function recovery. In a subsequent study, they tested human iPSC–secondary and tertiary NS [47]. As expected, the grafted cells differentiated and the animals showed significant locomotor recovery, supported by synapse formation between human iPSC-NS-derived neurons and host mouse neurons, the expression of neurotrophic factors, angiogenesis, axonal regrowth, and increased amounts of myelin in the injured area. Kobayashi and coworkers even transplanted iPSC–NSC into a primate model, which had positive results in terms of motor recovery and safety [42]. Nevertheless, in a long-term study with mice, the authors found that the grafted animals, which had initially recovered, gradually lost their motor function and developed tumors. Moreover, these tumors consisted of nestin-positive undifferentiated neural cells, and showed altered expressions of genes involved in the epithelial–mesenchymal transition, which may have promoted tumor invasion and the progression of grafted cells [48]. Fuhrmann and coworkers [51] developed an injectable hydrogel comprised of hyaluronan and methylcellulose to enhance the survival and differentiation of human iPS-OPC, and injected it in a subacute rat model of SCI. The transplanted animals formed teratomas; however, the hydrogel reduced the incidence to 50% compared to the group injected with medium. Okubo et al., 2016 tried to prevent tumorigenesis by pretreating human iPSC–NSC with γ-secretase inhibitor, which inhibits Notch signaling. This treatment promotes differentiation to neurons, resulting in improved motor function in subacute and chronic SCI models [53,60]. On the other hand, Itakura and coworkers [54] tested the efficacy of the inducible caspase9 gene in avoiding the tumorigenic transformation of human iPSC–NSC in vivo. They injected cells with a tumor formation tendency into a thoracic NOD-SCID mouse subacute model. iPSC–NSC were transplanted at the lesion epicenter and, once tumor formation was observed, a small-molecule chemical inducer of dimerization (CID) was administered to induce the apoptosis of the injected cells, and all grafted cells retreated. Before tumor formation, they found that cells were able to differentiate, and that mice showed improved hindlimb motor function until week 4 after transplantation, when tumors started appearing. In those animals on which the grafted cells were ablated, the motor function declined, although it remained slightly better than in sham mice. 

Recently, Chow and collaborators generated canine iPSC–NSC and transplanted them into pet dogs with chronic thoracic SCI after a traumatic accident. Observation via MRI did not show any changes in the lesion size or the glial scar of the animals. No adverse effects were observed either, demonstrating the safety of administering iPSC-derived NSC in dogs with follow-up for 6–12 months, particularly with respect to tumor formation at the injection site. However, the animals did not show an improvement in motor function [62].

Similarly to the progress made with human ESC, various groups have tried transplanting more differentiated human iPSC-derived cells. Hayashi and coworkers [41] used mouse iPSC to form neurospheres, which were subsequently differentiated to astrocytes. iPSC-derived astrocytes were transplanted into rats with thoracic SCI in the subacute phase of SCI. Although three different tests were used for locomotor evaluation (BBB test, inclined-plane test and SCANET MV-40 (Melquest), no significant improvement was detected in relation to the control group. Nevertheless, an increase in sensitivity to mechanical stimulus was described. Interestingly, Salewski and colleagues found that primitive NSC (incompletely committed to the neural lineage and still expressing some pluripotency markers) generated tumors after injection. By contrast, definitive NSC (committed to the neural lineage and with no expression of pluripotency markers) did not generate tumors [46]. These data indicate the relevance of transplanting more differentiated cells.

Using an induced thoracic contusion in NOD-SCID mice, Kawabata and collaborators grafted human iPSC–OPC into the lesion epicenter (subacute phase). The authors found that the grafted human iPSC–OPC contributed to remyelination, promoted axonal growth, and contributed to synapse formation with host mouse neurons, leading to enhanced functional recovery after SCI. However, when comparing the results with a previous study using human iPSC–NSC, they found no therapeutic differences in hindlimb motor function [52]. Recently, Patil and coworkers transplanted iPSC–pre-OPC into a model of chronic SCI. Their study consisted of ablating the glial scar to eliminate the hostile environment prior to cell transplantation. As a result, the lesion cavity was significantly reduced; nevertheless, no functional recovery was achieved. 

In summary, iPSC-derived cells have been shown to be efficacious in different animal models, especially in acute thoracic models (Table 1). Nonetheless, there are still some issues, such as their genetic and epigenetic abnormalities, immunogenic response, and tumorigenicity, which require caution when developing clinical-grade iPSC-derived cellular products. The Okano Laboratory group, which works in collaboration with Shinya Yamanaka, The Nobel Prize in Physiology or Medicine 2012, has been working in this regard, and achieved safe iPSC–NSC clones. As a result, the first human clinical study using iPSC-derived cells for subacute SCI is planned to be launched soon in Japan, with the primary objective of testing safety (Japan Registry of Clinical Trials (jRCT) number, jRCTa031190228) (Table 2). They plan to transplant human cGMP-grade iPSC–NSC, which have previously been shown to be safe both in vitro and in vivo. Four patients suffering C3/4-T10 level, complete subacute SCI will be recruited to be transplanted in the center of the injury with a dose of 2 × 10^6^ cells treated with γ-secretase inhibitor, to promote cell differentiation and decrease the risk of tumorigenesis. The efficacy of the treatment will be evaluated secondarily, assessing motor function following the International Standards for Neurological Classification of Spinal Cord Injury (ISNCSCI) in comparison with a historical control. Sensory function, spasticity, and quality of life will be assessed as well [70].

**Table 2:** Clinical trials for SCI using PSC-derived cell products.

Type of SCI	Clinical Trial Dentifier	PSC	Final Cell Type	References
Complete subacute SCI, ASIA Impairment Scale A. Last fully preserved neurological level from T-3 through T-11 (7 to 14 days following SCI)	NCT01217008 (ClinicalTrials.gov)	ESC	OPC (AST-OPC1)	[25]
Subacute cervical SCI, ASIA Impairment Scale A and B. Last fully preserved single neurological level from C-4 to C-7 (21 to 42 days following SCI)	NCT 02302157 (ClinicalTrials.gov)	ESC	OPC (AST-OPC1)	[25]
Complete subacute cervical (C4-C7) SCI, ASIA Impairment Scale A. (7 to 60 days following SCI)	NCT04812431 (ClinicalTrials.gov)	ESC	NSC (PSA-NCAM(+))	Not found
Complete subacute SCI (C3/4-Th10), ASIA Impairment Scale A (within 24 days following SCI)	jRCTa031190228 (Japan Registry of Clinical Trials)	Integration-free episomal iPSC from PBMC	NSC (to dopaminergic neuron fate)	[70]

SCI: spinal cord injury; PSC: pluripotent stem cells; ASIA: American Spinal Cord Injury Association; ESC: embryonic stem cells; iPSC-induced pluripotent stem cells; PBMC: peripheral blood mononuclear cells; OPC: oligodendrocyte progenitor cells; NSC: neural stem cells; PSA-NCAM: polysialylated form of neural cell adhesion molecule.

## 4. Conclusions

During the last 20 years of research with PSC-derived cells for SCI treatment, different animal SCI models have been assayed, using different types of PSC-derived cells, cultured under different conditions, grafted at different doses and time windows, and under different immunosuppression regimes (Table 1). These studies have provided new knowledge about the potential for SCI repair. However, the lack of standardization between studies complicates the comparison of results, hampering translation. A common strategy, as has been developed for Parkinson’s disease [71], would be desirable, and has already been suggested for SCI [72]. Yet, it can be said that the data collected from animal studies so far show, in general, satisfactory results for acute thoracic SCI models. Nevertheless, these preclinical studies have been ineffective in translating these results to humans, and few therapies for SCI with PSC-derived cells have reached the clinical stage (Table 2). 

To develop effective cures for SCI, it is necessary to obtain safe and fully characterized cells tested in a robust and repetitive SCI animal model that delivers, in every animal, a precisely measured force at the spinal cord. For initial experiments, validated and reproducible models in rodents such as MASCIS, Infinite Horizon or the Ohio Contusion device may be good options, but for therapy authentication, a model in large animals should be considered. Nevertheless, since human SCI is such a complex situation, it is very difficult to find a specific model that incorporates all variables. Therefore, the chosen model would depend mainly on the goals of each specific study [73]. The use of more predictive models is especially important for the development of therapies for neurological diseases, as the differences in the development of the central nervous system between species are marked. Human SCI models based on organoids have been developed [74,75], and can hopefully help complement the safety and efficacy of cell transplantation studies performed in animals, enhancing predictability and favoring translation. On the other hand, both in vivo and in vitro preclinical studies should include the gender dimension and evaluate age differences (reviewed [76]). Studies in aged individuals are appropriate, given the increasing incidence of SCI in elderly populations and their reduced recovery capacity. In this sense, studies including both sexes are increasing, and some articles using aged animals have been recently published [64,68]. 

Functional recovery assessments based on subjective evaluations or operator experience could be a red flag in many animal studies, and must be considered. An automated system for gait quantitative assessment would provide some parameters that can help standardize the evaluation of locomotion and forelimb function. Functional improvements should be measured by quantitative and objective protocols, such as the “Catwalk” automated quantitative gait analysis, among others [77].

The establishment of optimal protocols for immunosuppression is an important aspect commonly overlooked when designing allogeneic cell therapies. The classically used immunosuppressants (cyclosporine A, tacrolimus or rapamycin) are associated with important side effects in the host, and can affect the differentiation, proliferation and survival of transplanted cells [18,78,79,80,81]. These studies show that differences depend on the immunosuppressant, the concentration used, and the species. More studies are needed to clarify how immunosuppressants affect cell biology and optimize immunosuppression regimes for each specific cell therapy setting.

The precise type of cell(s), doses, and administration regimes required for significant sensory and motor function recovery in humans remain enigmatic. Furthermore, there is still no consensus on the preferred source of PSC, ESC or iPSC. Since ESC are derived from embryos, the use of ESC raises ethical concerns, and strict ethical committees regulate their use, complicating the development of ESC-based cell therapies [82]. Although iPSC circumvent the use of embryos, avoiding ethical problems, the extensive molecular manipulation and artificial conditions used during reprogramming may limit their use for regenerative medicine. In fact, many reports have highlighted iPSC safety and logistic concerns, including the retention of epigenetic memory of the cell of origin, mutation enrichment in oncogenes, and the prohibitive costs of autologous manufacturing (reviewed in [13,83]). Therefore, some scientists consider the use of ESC safer, and there are ongoing preclinical and clinical trials with ESC and iPSC sources for SCI and other pathologies [39]. The results of these studies will probably help clarifying which is the most optimal source for the development of large-scale therapies in humans. The banking of fully characterized HLA-matched/ablated ESC and iPSC lines produced under cGMP conditions will allow the use of the same lines in both preclinical and clinical studies, favoring standardization and fostering the development of valid therapeutic options for SCI patients in the future (reviewed in [84,85]).

Animal experiments with both ESC- and iPSC-derived products have shown that tumorigenic potential is an important safety issue. The PSC should be properly differentiated to avoid the transplantation of undifferentiated PSC that, due to their intrinsic self-renewal and differentiation potential, could form tumors (teratomas) in the host tissue [14]. The transplantation of additional differentiated cells alongside NSC and the use of cell-sorting techniques to remove remnant PSC could be interesting options to decrease tumorigenic risk. However, the safety of the PSC-derived final cell product must be thoroughly verified, using in vivo and in vitro models during the preclinical stages prior to first use in humans.

In general, PSC-derived cell therapies are recognized as one of the most promising therapeutic options for SCI repair, but there are still important challenges to overcome before they can be widely used in humans (Table 3). One of the main risks of PSC is their tumorigenic potential, although some preclinical and clinical studies have demonstrated that PSC-derived cells, when properly produced and controlled, are safe. Another problem to be solved is the high cost of producing PSC-derived medicinal products, and the absence of sufficiently relevant efficacy data (especially in chronic SCI) to justify the manufacture of these expensive therapies. The acute condition is more favorable for cell survival, but the organization of a clinical trial with acute patients is complex because the condition of the patient is not stabilized, and the neurologic status is still not completely established. On the other hand, treating chronic SCI patients may probably require scar removal and the filling of the cyst cavitation, as scar and cavitation are more evident in the late stages after SCI [86]. The use of combinational therapies, including cells, tissue scaffolds (to fill the cavitation area and promote axonal growth and cell migration), growth factors/anti-inflammatory agents (to increase cell survival), and enzymes (such as chondroitinase to disrupt the scar and favor cell grafting), can be approaches to consider for both acute and chronic SCI, to increase efficacy. 

## Figures and Tables

**Table 3:** Main challenges facing PSC-derived therapies for SCI.

1. PSC’ intrinsic tumorigenic potential
2. Immunogenicity problems associated with the allogeneic use (immunosuppressants required)
3. Optimal cell type, dose, route, timing and immunosuppression regime for each condition still not known
4. Lack of efficacy in some conditions, especially in chronic SCI
5. High production costs

## Data Availability

Data sharing is not applicable to this article.
